# Depression and associated factors among patients with type 2 diabetic mellitus in Ethiopia: a cross sectional study

**DOI:** 10.3389/fpsyt.2024.1454087

**Published:** 2024-10-30

**Authors:** Amanuel Dukato, Abera Beyamo, Aklilu Habte Hailegebireal, Wegayehu Zeneb Teklehaimanot, Abatwoy Ayfokru, Metages Alemnew, Daniel Birhanu Abate, Worku Abemie, Bethlehem Taye Mengistu, Yihenew Ayehu Dessie, Leweyehu Alemaw Mengstie, Bekahegn Girma

**Affiliations:** ^1^ School of Public Health, College of Medicine and Health Sciences, Wachemo University, Hossana, Ethiopia; ^2^ School of Nursing and Midwifery, Debre Berhan University, Debre Berhan, Ethiopia; ^3^ School of Medicine, Debre Berhan University, Debre Berhan, Ethiopia; ^4^ Department of Nursing, College of Medicine and Health Sciences, Bula Hora University, Bula Hora, Ethiopia

**Keywords:** depression, type 2 diabetic mellitus, factors, Ethiopia, psychopathology

## Abstract

**Background:**

Depression is a significant public health concern in both developed and developing countries. The burden of depression is particularly high among patients with chronic illnesses in developing countries, creating a dual challenge for both patients and the community. However, depression goes undiagnosed in 50%-75% of patients with chronic conditions such as diabetes mellitus. Additionally, there is limited information about the prevalence of depression among diabetic patients in Ethiopia. Therefore, this study aimed to assess the prevalence and associated factors of depression among type 2 diabetic patients in Ethiopia.

**Method:**

An institution-based cross-sectional study was conducted on 376 randomly selected type 2 diabetic patients. Data were collected through face-to-face interviews and from patients’ follow-up records. The data were entered into EpiData version 4.6 and analyzed using STATA 14. Bivariable and multivariate logistic regression analyses were employed to identify associated factors. Variables with a p-value of less than 0.25 in the bivariable analysis were selected for multivariate logistic regression. Model fitness was assessed using Hosmer-Lemeshow’s test, and associations were reported using adjusted odds ratios with 95% confidence intervals

**Results:**

In this study, the prevalence of depression among type 2 diabetic patients was found to be 69.72% (95% CI: 64.75, 74.27). Three factors were significantly associated with depression in these patients: the duration of diabetes mellitus since diagnosis [AOR: 1.17; 95% CI (1.02, 1.34)], glycaemic control [AOR: 1.8; 95% CI (1.09, 3.01)] and cigarette smoking [AOR: 2.18; 95% CI (1.07, 4.46)].

**Conclusion:**

The prevalence of depression among type 2 diabetic patients was high. The Federal Ministry of Health, stakeholders, and the Ethiopian Diabetes Association should collaborate to reduce this burden. Mental health assessment and treatment should be integrated into chronic care follow-up services. Additionally, healthcare providers should closely monitor and counsel patients who smoke and those with poor glycemic control.

## Background

Depression is a prevalent mental condition characterized by persistent sadness, anhedonia, fatigue, changes in appetite, disinterest in daily activities, feelings of guilt, low self-esteem, sleep disturbances, exhaustion, and suicidal thoughts (1). It significantly impacts individuals and the country due to its disruption of daily life (2, 3). If left untreated, depression can worsen and persist; however, treatments are often very effective in alleviating symptoms (4).

Diabetes is a chronic condition where the body either does not produce sufficient insulin, produces no insulin at all, or cannot effectively use the insulin it does produce. Type 2 diabetes mellitus (T2DM) typically develops in adulthood (5, 6). The global prevalence of diabetes among individuals aged 20 to 79 is 9.3%, with 79.4% of cases occurring in low- and middle-income countries (7, 8). Recent studies indicate that 28% of T2DM patients globally, 24% in Europe, 29% in Australia, 32% in Asia, and 27% in Africa experience concurrent depression (9, 10). In Ethiopia, the prevalence of depression among T2DM patients ranges from 13% to 40.4%, reflecting variations in study duration, settings, and assessment tools (11).

Depression and diabetes mellitus (DM) have a bidirectional relationship, with early-life depression increasing the risk of developing diabetes, and diabetes subsequently raising the likelihood of depression later in life (12). Individuals with DM are at a heightened risk for depression (13). The mechanism of their interaction is expressed like depression can be stimulated by hyperglycaemia and dyslipidaemia because they worsen inflammation and lower serotonin levels in the brain. Disrupted insulin signaling in T2DM can decrease glycaemic control, and depression can affect insulin signing. Prolonged stress in individuals with depression further exacerbate type 2 diabetes by increasing cortisol levels, which in turn causes insulin resistance and accelerates the development of T2DM (14–16).

The intricate relationship between mental health and illness treatment is highlighted by recent developments in our understanding of the psychological problems related to diabetes mellitus (17, 18). Diabetes care now includes psychological issues such as melancholy, anxiety, and diabetes distress as essential components (19–21). According to research, the constant demands of self-care, particularly worries about complications, cause significant mental discomfort for people with diabetes (18, 22). The Diabetes Distress Scale is one of the instruments used in modern clinical practice to provide prompt and focused mental health interventions (14–16, 23, 24). Furthermore, patients who get psychological assistance are better able to control their anxiety of hypoglycemia (15, 25).

Technological advancements have made diabetic psychological concerns management even more advanced. Research demonstrates that these digital methods of delivering cognitive-behavioral therapy, mindfulness-based therapies, and acceptance and commitment therapy are beneficial in reducing diabetes-related suffering and enhancing mental health (26–29).

The coexistence of diabetes and depression can lead to poor treatment adherence, poor glycaemic control, higher complication rates, reduced quality of life, increased healthcare utilization and costs, greater disability, loss of productivity, and an elevated risk of death (30–33). It also heightens the risk of both micro vascular and macro vascular complications (8, 34). Moreover, it has work related risks like sickness absence, unemployment and reduced income (35).

Preceding studies identified age, smoking habit, being women, poor social support, number of comorbidities, higher level of cholesterol, physical disability and body mass index as risk factors for depression among diabetic adults (36–38).

In the developed world, numerous studies have been conducted to assess the prevalence and determinants of depression among diabetic patients. However, fewer studies have been carried out in Africa, including Ethiopia. Therefore, this study aimed to assess the prevalence and associated factors of depression among patients with T2DM in Ethiopia.

## Materials and methods

### Study period and setting

The study was conducted from June 30 to July 30, 2022, in comprehensive specialized hospitals located in Southern Ethiopia. These hospitals include Wachamo University Comprehensive Specialized Hospital (WUCSH), Wolaita Sodo University Comprehensive Specialized Hospital, and Worabe Comprehensive Specialized Hospital. Our study took place at WUCSH, situated in Hosanna Town, Hadiya Zone, Southern Ethiopia, approximately 232 kilometres from Addis Ababa, the capital city of Ethiopia.

WUCSH provides general and specialized healthcare services, along with teaching and research activities. It offers comprehensive diabetes-related services, with the diabetic clinic being one of the hospital’s oldest clinics, providing care and follow-ups for patients with all types of diabetes.

### Study design

An institutional based cross-sectional study.

### Population

#### Source population

All adult patients with T2DM who have follow-up in comprehensive specialized hospitals found in southern Ethiopia.

### Study population

All adult patients diagnosed with type 2 Diabetes Mellitus receiving follow-up care at the selected hospital (WUCSH).

### Eligibility criteria

All adult patients (>18 years old) diagnosed with type 2 Diabetes Mellitus who were receiving care at WUCSH and provided consent were included. However, patients who were currently taking antidepressant medication for their depressive symptoms, those who were severely ill during the data collection period, and newly diagnosed type 2 diabetes mellitus patients at the time of data collection were excluded.

### Sample size determination

The sample size was determined using the single population proportion formula, based on the following assumptions: a prevalence (p) of depression among adult patients with Diabetes Mellitus (37%), as reported in a previous study conducted in Ethiopia (39), a confidence level (Zα/_2_) of 95%, and a margin of error of 5%.


$$n=\frac{{\left(\frac{Z\alpha }{2}\right)}^{2}P\left(1-P\right)}{d^{2}}$$

After adding 5% non-response rate, the minimum required sample size was 376.

### Sampling technique and sampling procedure

Before data collection, the total number of diabetic patients who had visited the hospital since 2021 was obtained from patient records. The average number of diabetic patients visiting the hospital over a one-month period was then calculated to be 1071. Participants for interviews were selected using systematic random sampling. The sampling interval (k) was determined by dividing the expected number of diabetic patients having a follow-up visit at the time of data collection by the calculated sample size (k = 3). Starting from a random number between one and three, eligible individuals were interviewed at every third interval based on the order of their clinical evaluation.

### Operational definitions

Depression: if patients Patient Health Questionnaire (PHQ) score was 5 or higher (38).

Poor glycaemic control: having an average fasting blood sugar (FBS) level greater than 130 mg/dl over a three-month period (40).

Regular physical activity: defined as engaging in exercise for at least 30 minutes or walking for 3 or more days per week (40).

Current cigarette smokers: individuals who had smoked cigarettes within the 30 days preceding the data collection (35).

### Data collection procedure and quality assurance

A semi-structured questionnaire was utilized to collect data through face-to-face interviews and record reviews. The questionnaire was originally designed in English and translated into Amharic, then back-translated into English to ensure consistency.

A pre-test was conducted on 5% of the sample size to assess language clarity, appropriateness of data collection tools, time estimation, and necessary adjustments based on the feedback. Data collectors and supervisors underwent comprehensive training on the data collection tool and process. Subsequently, investigators and supervisors meticulously reviewed all collected data for completeness and consistency. Random samples of questionnaires were checked for inconsistencies, coding errors, completeness, clarity, and any missing values before data entry.

The PHQ tool is 9-item depression screening and diagnostic questionnaire. Each question requires participants to rate the frequency of depressive symptoms experienced during the two weeks prior to evaluation. These items include: 1) loss of interest, 2) depressed mood, 3) insomnia or hypersomnia, 4) fatigue or loss of energy, 5) appetite disturbances, 6) guilt or worthlessness, 7) diminished ability to think or concentrate, 8) psychomotor agitation or retardation, and 9) suicidal thoughts. Scores for each item range from 0 (“not at all”) to 3 “nearly every day” with a total score ranging from 0 to 27 (41).

The PHQ tool was previously validated in a study conducted in East Africa, which reported a Cronbach’s alpha of 0.85. Furthermore, its reliability was found to be 0.92 (42). Additionally, the Amharic version of the PHQ was specifically validated in a study conducted in Ethiopia, where the kappa agreement and interclass correlation coefficient were 0.80 and 0.92, respectively (43). Even though the Amharic version had already been validated in earlier studies, in this study the Cronbach’s alpha was also 0.79.

### Data analysis

The data were entered using EpiData version 4.6.0.2 and exported to STATA version 14 for analysis. Descriptive statistics such as frequencies, means, and standard deviations were computed to characterize the variables.

Logistic regression analysis was conducted to identify factors associated with depression. Variables with a p-value less than 0.25 in the bivariable analysis were included in the multivariable logistic regression model. The Hosmer-Lemeshow’s test was performed to assess the goodness of fit of the model. In the multivariable logistic regression model, adjusted odds ratios with 95% confidence intervals were calculated to determine the strength and direction of associations.

## Results

In the current study, a total of 360 respondents were participated with a response rate of 95.7%.

### Socio-demographic characteristics of respondents

The respondents had a mean age of 47.2 years, with a standard deviation of ±15.7. Of the participants, 210 (58.3%) were male and identified as Protestant. Approximately seventy-two percent of the respondents were married, and 161 (44.7%) had completed a diploma or higher education. Lastly, 102 (28.3%) of the respondents were self-employed (**Table 1**).

**Table 1:** Socio-demographic characteristics of type 2 diabetic patients included in this study, 2024 (n = 360).

Characteristics	Category	Frequency (%)
Sex	Male	210 (58.3)
	Female	150 (41.7)
Residence	Urban	168 (46.7)
	Rural	192 (53.3)
Marital Status	Single	58 (16.1)
	Married	261 (72.5)
	Divorced	19 (5.3)
	Widowed	22 (6.1)
Educational status	No formal education	76 (21.1)
	Primary	43 (11.9)
	Secondary	80 (22.2)
	Diploma and above	161 (44.7)
Occupation	Government Employed	94 (26.1)
	Self employed	102 (28.3)
	Farmer	65 (18.1)
	Student	25 (6.94)
	Housewife	53 (14.7)
	Others	21 (5.8)
Religion	Protestant	210 (58.3)
	Orthodox	81 (22.5)
	Muslim	48 (13.3)
	Catholic	12 (3.3)
	Others	9 (2.5)

### Clinical factors associated with the risk of depression

Two hundred three (56.4%) of the participants were receiving non-insulin treatment, and one hundred (27.8%) of them were also diagnosed with hypertension. The mean fasting blood sugar (FBS) level among respondents was 197.1 mg/dl (± SD: 85.0), and the average duration of diabetes mellitus (DM) was 3.1 years (± SD: 2.1). One hundred eighty-eight (52.2%) of the respondents achieved good glycaemic control, and the majority (83.1%) did not have a family history of DM (**Table 2**).

**Table 2:** Clinical characteristics of type 2 diabetic patients included in this study, 2024 (n = 360).

Characteristics	Category	Frequency (%)
DM treatment	Insulin	53 (14.7)
	Non-insulin	203 (56.4)
	Combined	104 (28.9)
Glycaemic control	Good	188 (52.2)
	Poor	172 (47.8)
Hypertension	Yes	100 (27.8)
	No	260 (72.2)
Family history of DM	Yes	61 (16.9)
	No	299 (83.1)
Body mass index	Normal	283 (78.6)
	Overweight	77 (21.4)
DM complications	Yes	123 (34.2)
	No	237 (65.8)

### Behavioral factors associated with the risk of depression

Three hundred thirty-three (92.5%) of the participants did not engage in regular exercise. Forty-seven (13.1%) of them reported being smokers. Finally, approximately one-fifth (20.3%) of our respondents experienced sexual dysfunction (**Table 3**).

**Table 3:** Behavioral related characteristics among type 2 diabetic patients included in this study, 2024 (n = 360).

Characteristics	Category	Frequency (Percent)
Exercise	No	333 (92.5)
	Yes	27 (7.5)
Cigarette smoking	Yes	47 (13.1)
	No	313 (86.9)
Alcohol	Yes	47 (13.1)
	No	313 (86.9)
Chat chewing	Yes	30 (8.3)
	No	330 (91.7)
Sexual dysfunction	Yes	73 (20.3)
	No	287 (79.7)

### Prevalence of depression among T2DM patients

The prevalence of depression among diabetic patients was 69.72% (95% CI: 64.75, 74.27).

### Factors associated with depression among people with T2DM

Gender, marital status, duration of diabetes mellitus since diagnosis, hypertension, family history of diabetes mellitus, adherence to exercise, body mass index, diabetes mellitus complications, alcohol consumption, glycaemic control, chat chewing, and cigarette smoking status were considered as candidate variables for multivariable logistic regression analysis. However, in the multivariable logistic regression analysis, only three variables duration of diabetes, glycaemic control, and cigarette smoking showed a significant association with depression among type 2 diabetic patients (**Table 4**).

**Table 4:** Bivariable and Multivariable analysis to identify factors associated with depression among people with T2DM in Ethiopia, 2024 (n= 360).

Characteristics	Category	COR (95% CI)	AOR (95% CI)
Sex	Male	1	1
	Female	0.74 (0.47, 1.16)	0.67 (0.40, 1.13)
Marital status	Single	1	1
	Married	0.67 (0.35, 1.28)	0.69 (0.34, 1.40)
	Divorced	6.28 (0.77, 51.16)	3.14 (0.95, 7.97)
	Widowed	1.57 (0.46, 5.38)	1.08 (0.77, 2.28)
DM duration	< 5years	1	1
	≥ 5 years	1.15 (1.02, 1.29)	1.17 (1.02, 1.34)*
Hypertension	Yes	1	1
	No	1.63 (1.00, 2.65	1.23 (0.57, 2.65)
Family history of DM	Yes	1	1
	No	0.64 (0.34, 1.22)	0.68 (0.32, 1.43)
DM complication	No	1	1
	Yes	1.745 (1.09, 2.78)	1.78 (0.84, 3.76)
Glycaemic Control	Good	1	1
	Poor	1.51 (0.96, 2.36)	1.81 (1.09, 3.01)*
Cigarette smoking	No	1	1
	Yes	1.86 (0.99, 3.49)	2.18 (1.07, 4.46)*
Exercise	No	1	1
	Yes	0.17 (0.04, 0.73)	0.29 (0.06, 1.44)
Alcohol	Yes	1	1
	No	0.50 (0.23, 1.08)	0.62 (0.24, 1.57)
Chat chewing	Yes	1	1
	No	0.43 (0.16, 1.17)	0.48 (0.15, 1.518)
Body mass index	Normal	1	1
	Overweight	0.70 (0.41, 1.19)	0.63 (0.33, 1.18)

The bold values indicate significantly associated factors.

* indicates significantly associated factors.

Therefore, this study supports the association between clinical and psychosocial factors with depression among diabetic patients. However, not support the hypothesis that declares the connection between socioeconomic factors and depression among diabetic patients.

In this study, Type 2 diabetic patients with poor glycaemic control had 1.8 times higher risk of depression compared to those with better control [AOR: 1.8; 95% CI (1.09, 3.01)]. Patients with a longer duration since diabetes diagnosis had increased odds of depression [AOR: 1.17; 95% CI (1.02, 1.34)]. Lastly, diabetic patients who smoked cigarettes were at 2.1 times higher risk of depression compared to non-smokers [AOR: 2.18; 95% CI (1.07, 4.46)].

## Discussion

The current study aimed to determine the prevalence of depression and its contributing factors among patients with T2DM diabetes mellitus in Ethiopia. The prevalence of depression was 69.72% (95% CI: 64.75, 74.27).

This finding was in line with studies conducted in Egypt (44) and Iran (45). This might be due to similarity in diagnostic tool and study design. However, the prevalence was higher than studies conducted in Sudan (46), India (47), Vietnam (48), Saudi Arabia (49) and Ethiopia (38, 39, 50–52). This variation may be attributed to differences in sample size, study settings, and assessment tools. Additionally, it could be influenced by changes in environmental, cultural, and social contexts, as well as disparities in healthcare service availability and quality. Lastly, the prevalence reported in our study was low as compared to study conducted in Tanzania (13). This might be due to difference in health care seeking behavior and availability of psychiatric services for patients with chronic diseases.

Diabetic patients who had poor glycaemic control had more likely to manifest depression. This finding was supported by studies conducted in Ghana (53), Iran (54), China (55) and Peru (56). This could be attributed to poor glycaemic control, which increases levels of brain neurotransmitters linked to depression and alters connections between brain regions that regulate emotions (57). Additionally, it may be related to the fact that lower HbA1C levels are associated with a reduced risk of diabetic complications (58).

The duration of diabetes mellitus since diagnosis was also found to be positively associated with depression. This finding is consistent with studies conducted in Ethiopia (39). This could be because a longer duration of diabetes mellitus is associated with an increased risk of developing diabetes-related complications and comorbidities, which may explain the higher likelihood of depression with longer illness duration.

Finally, diabetic patients who smoke cigarettes were found to have a higher risk of depression. This finding is consistent with a study conducted in Tanzania (13). This may be because smoking can alter neurocircuitry, increasing sensitivity to environmental stressors and thereby raising the risk of developing depression.

This study had several limitations. Firstly, due to its cross-sectional design, establishing the temporal relationship between dependent and independent variables was challenging. Secondly, important factors such as intimate partner violence, food security, and social support were not assessed.

## Conclusion

In the current study, the prevalence of depression among diabetic patients was found to be higher compared to other studies. Additionally, three factors duration of the disease, poor glycaemic control, and cigarette smoking were identified as independent risk factors for depression among patients with type 2 diabetes mellitus.

To fill this gap, the Federal Ministry of Health, stakeholders, and the Ethiopian Diabetic Association should collaborate and design new strategies like integrating the mental health assessment and treatment service into chronic follow-up clinics. Moreover, in future innovative technologies like mHealth and telemedicine as well as cognitive behavioral therapy should be considered and implemented to decrease the burden of depression.

Furthermore, mindfulness-based interventions, and acceptance and commitment therapy should be delivered through digital technologies to increase awareness and self-care practice skills. Healthcare providers should also prioritize and provide counseling to patients who smoke and those with poor glycaemic control. Lastly, future researchers should conduct longitudinal studies to gain a deeper understanding of this issue.

## Data Availability

The raw data supporting the conclusions of this article will be made available by the authors, without undue reservation.
